# Clinico-Radiological-Pathological Correlation in a Rapidly Evolving Well-Differentiated Orbital Liposarcoma: A Case Report

**DOI:** 10.7759/cureus.77674

**Published:** 2025-01-19

**Authors:** Rikako Iwasaki, Yoshiyuki Kitaguchi, Shusuke Hayashi, Takeshi Morimoto, Kohji Nishida

**Affiliations:** 1 Department of Ophthalmology, Higashiosaka City Medical Center, Osaka, JPN; 2 Department of Ophthalmology, Osaka University Graduate School of Medicine, Osaka, JPN; 3 Department of Ophthalmology, Kinan Hospital, Wakayama, JPN; 4 Department of Advanced Visual Neuroscience, Osaka University Graduate School of Medicine, Osaka, JPN

**Keywords:** dedifferentiation, exenteration, mri, tumor progression, well-differentiated orbital liposarcoma

## Abstract

Rapidly progressing orbital liposarcomas, while rare, pose significant diagnostic challenges due to their varied clinical and radiological presentations. A 76-year-old female presented with a suspected well-differentiated orbital liposarcoma 16 months after the onset of proptosis and diplopia. Initial magnetic resonance imaging (MRI) revealed a homogeneous, high-intensity mass in the left superior orbit. Although oral corticosteroids were administered, the patient’s condition worsened over the following 13 months, with subsequent MRI revealing a heterogeneous mass. Orbital exenteration was performed, and histopathological analysis confirmed the diagnosis of a well-differentiated liposarcoma despite the rapid progression and imaging changes.

This case highlights that rapid clinical and radiological changes in orbital liposarcomas do not necessarily indicate dedifferentiation. The discrepancy between imaging progression and histopathological findings emphasizes the critical role of pathological evaluation in making a definitive diagnosis. Treatment decisions, including aggressive surgical approaches, should be based on a comprehensive assessment of the clinical presentation, imaging features, and histopathological characteristics tailored to the individual patient's condition and disease progression.

## Introduction

Liposarcoma is one of the most common soft tissue sarcomas, accounting for approximately 20% of all soft tissue malignancies. These tumors arise from adipose tissue and can occur in various anatomical locations, with the extremities and retroperitoneum being the most frequent sites [1]. Among these locations, orbital occurrence is extremely rare, with only approximately 80 cases reported in the English literature since the first description by Strauss in 1911 [2,3]. The clinical presentation typically includes proptosis, often accompanied by symptoms such as diplopia, lid swelling, ptosis, reduced visual acuity, subconjunctival mass, or pain [3-5]. The anatomical location of orbital liposarcomas poses unique challenges for surgical intervention due to the confined orbital space and the proximity to vital structures like the optic nerve and extraocular muscles. Consequently, complete resection without exenteration is often difficult, complicating treatment approaches.

Liposarcomas are categorized into five subtypes: well-differentiated, myxoid, pleomorphic, myxoid pleomorphic, and dedifferentiated [6,7]. In orbital liposarcomas, the well-differentiated and myxoid types predominate [4]. These liposarcomas are typically managed with debulking surgery owing to favorable survival prognosis [8]. In contrast, dedifferentiated liposarcomas often require more aggressive intervention, such as exenteration, given their association with more aggressive behavior and poorer prognosis [9]. This prognostic difference significantly affects treatment planning, making accurate subtype classification through pathological evaluation crucial.

Despite their generally favorable prognosis, well-differentiated orbital liposarcomas have the potential to transform into more aggressive dedifferentiated forms. Early detection of this malignant transformation is crucial for effective treatment planning. However, distinguishing between stable, well-differentiated tumors and those undergoing dedifferentiation remains challenging. To date, three cases of confirmed malignant transformation from well-differentiated to dedifferentiated orbital liposarcomas have been reported [10-12], all of which showed rapid clinical and radiological changes. However, the specific indicators of this progression are still unclear, complicating clinical decision-making, particularly regarding the optimal timing for aggressive surgical intervention.

Here, we present a case of a well-differentiated orbital liposarcoma that demonstrated rapid clinical and radiological progression, raising concerns about dedifferentiation. However, histopathological analysis revealed the maintenance of well-differentiated features. By detailing the correlation between magnetic resonance imaging (MRI) findings and pathological features, we aim to enhance our understanding of the progression spectrum in well-differentiated orbital liposarcomas.

## Case presentation

This case report adheres to the CAse REport (CARE) guidelines [13]. This study was approved by the Institutional Review Board of Osaka University Hospital (approval number 23212) and adhered to the Declaration of Helsinki. The patient provided written informed consent to publish this report and the accompanying images after we explained the process of ensuring patient anonymity.

**Figure 1:**
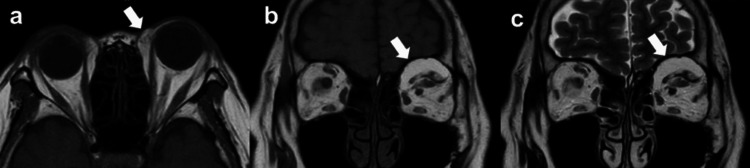
MRI images taken at the first visit to the previous hospital. a, b. Axial and coronal T1-weighted MRI images showing a homogeneous, high-intensity mass involving the superior oblique muscle in the superior orbit (arrow); c. Coronal T2-weighted MRI image showing a high-intensity mass (arrow).

A 76-year-old female patient was referred to our institution with a suspected well-differentiated orbital liposarcoma. She had no medical history of systemic diseases other than a history of treatment for chronic sinusitis when she was young and prior cataract surgery in both eyes. Approximately 16 months before referral, the patient presented to another hospital with a two-month history of proptosis and diplopia. Physical examination revealed left proptosis (19 mm measured with a Hertel exophthalmometer) with eyelid swelling and restricted eye movement during upward gaze. Her visual acuity (VA) was 20/20, and the left eye’s intraocular pressure (IOP) was 20 mmHg. MRI revealed a 30×10×28 mm tumor in the left superior orbit, primarily located in the preaponeurotic fat pad and extending around the superior oblique muscle. T1-weighted (Figures 1a, 1b) and T2-weighted (Figure 1c) images showed a homogeneous, high-intensity mass in the superior orbit. Based on these findings, the patient was diagnosed with suspected idiopathic orbital inflammatory syndrome and treated with oral corticosteroids tapered over two months. Although this anti-inflammatory treatment slightly decreased the left upper eyelid swelling, the left proptosis persisted. Thereafter, at the patient's request, monthly follow-up was initiated without treatment.

**Figure 2:**
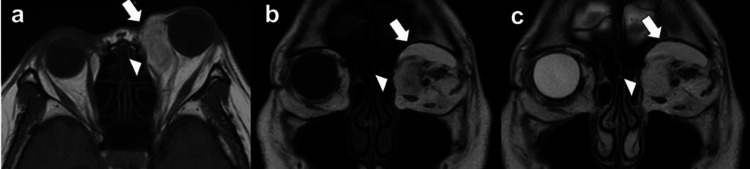
MRI images taken at the previous hospital 13 months after the first visit. a, b. Axial and coronal T1-weighted MRI images showing a homogeneous, high-intensity mass (arrow) and a newly developed heterogeneous appearance with mixed low and high-signal intensities (arrowhead); c. Coronal T2-weighted MRI image also showed both homogeneous area (arrow) and heterogeneous area (arrowhead) in the superomedial orbit.

Approximately 13 months after her first visit, the patient returned to the previous hospital with worsening symptoms. Examination revealed rapid progression of the left proptosis, inferolateral deviation of the eyeball, and severely restricted ocular motility. Her left upper eyelid showed significant redness and swelling, and she could not open the eyelid. A prominent corneal epithelial defect was noted. VA was slightly reduced, and the left eye’s IOP was 24 mmHg. The second MRI at the 13-month follow-up (Figure 2) revealed an enlarged tumor measuring 34×21×38 mm and compressing the eyeball. The tumor had extended medially, involving the superior oblique muscle, and inferiorly, involving the inferior rectus muscle, and posteriorly, approaching the orbital apex. The original lesion in the preaponeurotic fat pad maintained a homogeneous high intensity on T1 and T2-weighted images and a low intensity on Short Tau Inversion Recovery (STIR) images (Figures 2a-2c), consistent with the initial MRI. However, a newly developed lesion in the superomedial orbit showed a heterogeneous appearance with mixed signal intensities on T2-weighted images (Figures 2a-2c). A transconjunctival incisional biopsy was performed, and the patient was referred to our institution with a suspected pathological diagnosis of well-differentiated liposarcoma. Subsequently, the symptoms gradually worsened, and VA deteriorated significantly.

**Figure 3:**
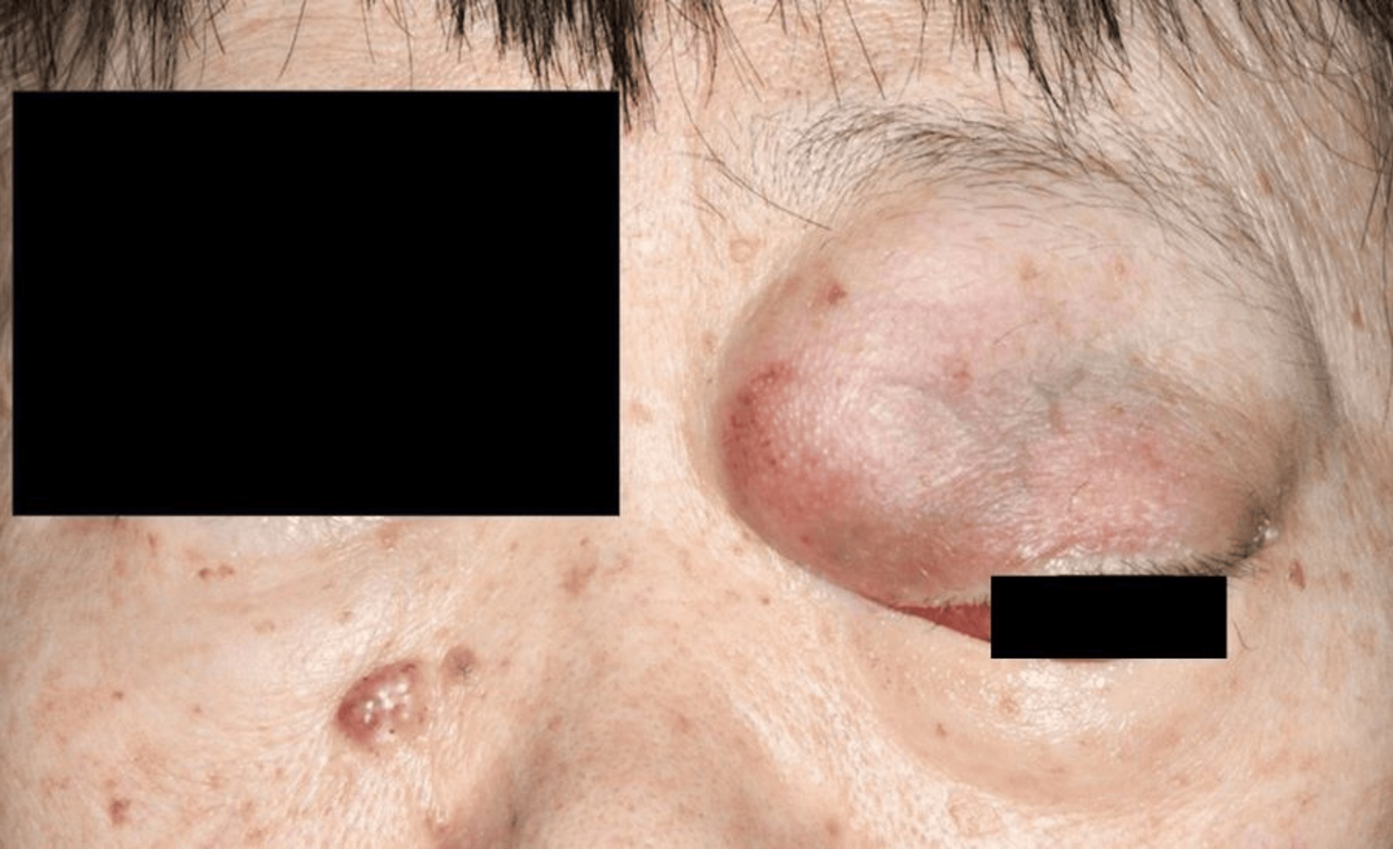
Clinical photographs at the first visit to our institution. A large tumor is visible in the left eye, with the eyeball deviating inferolaterally.

Upon presentation to our institution, the patient had marked proptosis, and inferolateral deviation of the left eye was prominent (Figure 3). VA was 20/13 and 20/400 in the right and left eye, respectively, with IOPs of 12.5 mmHg in the right eye and 23 mmHg in the left. A computed tomography scan of the whole body ruled out systemic metastases but confirmed the presence of a hypodense lesion in the superomedial orbit. Considering the possibility of malignant transformation due to the rapid tumor growth and changes in MRI findings, orbital exenteration was performed under general anesthesia. A lid-sparing exenteration technique was employed. Careful dissection between the dermis and orbicularis oculi muscle in the upper eyelid was performed to avoid disturbing the orbital septum because the tumor existed within the orbital septum of the upper eyelid. For the other areas, including the lower eyelid and lateral canthus, the dissection was performed beneath the orbicularis oculi muscle. Following the removal of orbital contents, the surgical margin at the orbital apex was examined histologically to confirm the absence of tumor cells. Histopathological examination revealed distinct features consistent with the imaging findings.

**Figure 4:**
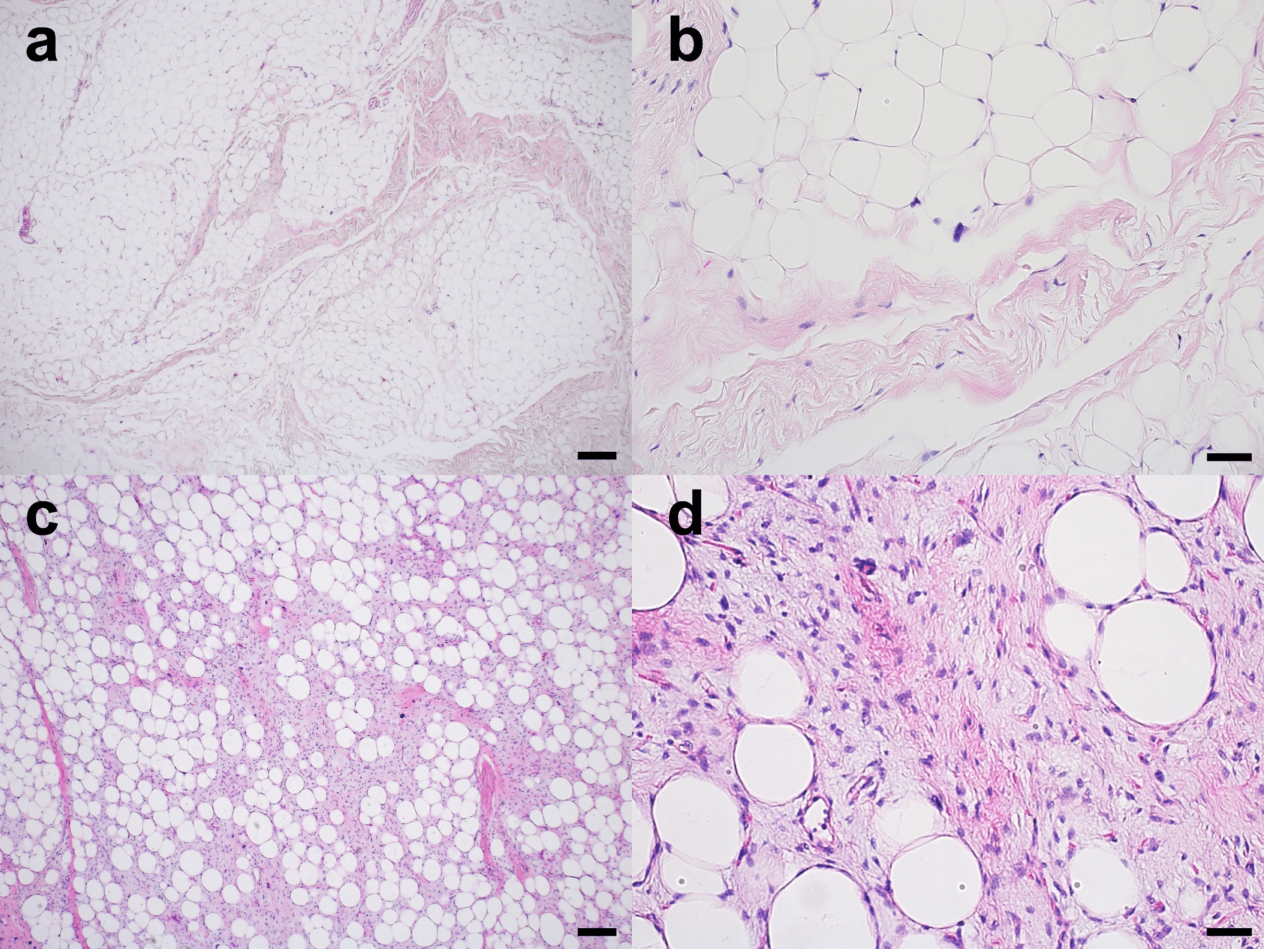
Histopathological findings (hematoxylin-eosin stain). Multinucleated stromal cells and atypical adipocytic cells are observed within fibrous bands. a, b. Region showing homogeneous signals on T1 and T2-weighted MRI predominantly consists of differentiated adipocytes; c, d. Newly developed region showing heterogeneous signals on T1 and T2-weighted MRI, primarily consists of adipocytes with a significantly increased proportion of atypical stromal cells within fibrous septa. Scale bars=200 μm (a, c); 50 μm (b, d).

The region that appeared homogeneous on T1 and T2-weighted images was predominantly composed of differentiated adipocytes, with only a small proportion of atypical stromal cells (Figures 4a, 4b). In contrast, the newly developed region, which showed heterogeneous signals on T1 and T2-weighted images, displayed a more complex composition. Although this area was also largely composed of adipocytes, it contained a noticeably increased proportion of atypical stromal cells within the fibrous septa (Figures 4c, 4d).

**Figure 5:**
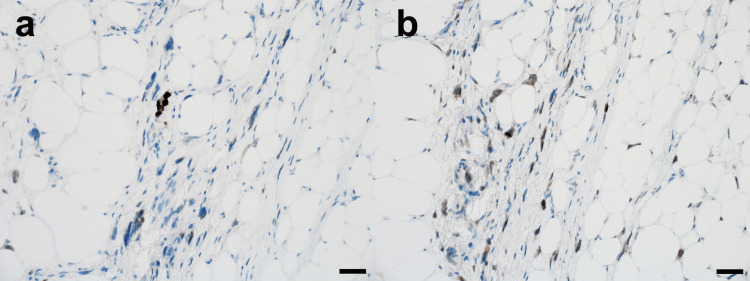
Histopathological findings (immunostaining). Region with heterogeneous MRI signals. a. MDM2-positive staining; b. CDK4-positive staining. Scale bar=50 μm.

These cells were large, had hyperchromatic nuclei, and showed signs of active proliferation. Immunohistochemically, the tumor cells were positive for p16, mouse double minute 2 (MDM2) (Figure 5a), and cyclin-dependent kinase 4 (CDK4) (Figure 5b). Ki-67 positivity was observed in as few as 10% of the cells. Despite the changes in the pathological findings compared to the previously existing lesion, the diagnosis remained well-differentiated liposarcoma due to the maintained adipocytic differentiation and low Ki-67 index (≤10%). 

At the four-month follow-up, the patient showed no signs of infection, local recurrence, lymph node enlargement, or metastasis.

## Discussion

We present a case of well-differentiated orbital liposarcoma that exhibited rapid progression and suspected dedifferentiation. The sudden exacerbation of symptoms, including worsening proptosis and ocular deviation, along with heterogeneous changes in MRI findings, matched the clinical presentations previously reported in dedifferentiated cases [10-12]. This was particularly concerning given that dedifferentiation occurs in 5-15% of well-differentiated liposarcomas [9,14], typically developing over an average latent period of 7.7 years [12,15]. However, histopathological evaluation in our case revealed an important and unexpected finding. Despite the aggressive clinical course and imaging changes, the tumor remained a well-differentiated liposarcoma without evidence of dedifferentiation. This case demonstrates that rapid clinical changes and alterations in MRI findings in well-differentiated orbital liposarcomas can occur without histopathological evidence of dedifferentiation, highlighting the importance of careful interpretation of clinical and radiological progression.

MRI findings for well-differentiated orbital liposarcomas vary widely in the literature. Some reports describe them as homogeneous, with high intensity on T1 and T2-weighted images and low intensity on STIR sequences [4,16], while others have noted heterogeneous appearances with relatively low-intensity signals [3,4]. Our case clearly demonstrates both features: the initial lesion in the preaponeurotic fat pad showed homogeneous high-intensity signals, whereas the later developed superomedial orbital lesion displayed heterogeneous signals. This observation suggests that the MRI findings of well-differentiated liposarcomas change depending on the stage of disease progression. Furthermore, our findings challenge the conventional interpretation that the development of fatty components into non-fatty solid components on MRI indicates dedifferentiation. Instead, we found that these imaging features can represent disease progression within the well-differentiated entity [12,17,18]. Although not performed in this case, gadolinium-enhanced T1-weighted MRI might provide additional insights, as increased enhancement has been associated with dedifferentiation [10].

Pathological analysis revealed an increased proportion of atypical stromal cells and a decreased proportion of differentiated adipose tissue in the newly developed lesion. Variations in the extent of malignancy depending on location have been previously reported [19]. Genetically, well-differentiated and dedifferentiated liposarcomas are associated with amplification of 12q13-15 [13]. Key genes in this region, including MDM2, cyclase-associated protein (CPM), and solute carrier family 35 member E3 (SLC35E3), are significantly amplified and play a critical role in the progression of dedifferentiated liposarcoma. Recent genomic analyses have further clarified the molecular pathways involved in the transition from well-differentiated to dedifferentiated types [20].

In the present case, both lesions were diagnosed as well-differentiated liposarcomas based on the presence of abundant differentiated adipose tissue and the absence of rapid mitotic activity, with a low Ki-67 labeling index (below 10%). These findings did not meet the criteria for dedifferentiated liposarcoma, which typically exhibits non-uniform fibroblastic spindle cells with nuclear atypia, moderate cellularity, and loss of differentiated adipocytes [11]. The diagnosis of liposarcoma was further supported by positive immunohistochemical staining for MDM2 and CDK4, which are established markers of this neoplasm [10].

Management options for orbital liposarcoma include complete surgical resection, radiotherapy, and chemotherapy [3]. Surgical excision is generally preferred due to the frequent resistance of liposarcomas to other treatments [9]. Studies have shown that initial orbital exenteration results in a significantly lower recurrence rate (9%) than subtotal resection (67-74%) [3,4,9]. Additionally, infiltration of the superior oblique and rectus muscles makes complete resection with globe-sparing difficult. Considering the risk of future dedifferentiation, which increases the likelihood of metastasis in 10-15% of cases and decreases 5-year survival to 70%, orbital exenteration was chosen as the most appropriate treatment [9]. This aggressive approach aims to minimize the risk of recurrence and improve the long-term outcomes in patients with this challenging malignancy.

## Conclusions

This case demonstrates that rapid symptom progression and imaging changes in well-differentiated orbital liposarcomas do not always signify a transformation to the dedifferentiated subtype. Notably, while the rapidly growing lesion displayed fewer adipocytes and an increased proportion of atypical stromal cells compared to the original tumor, it still maintained the overall histological characteristics of a well-differentiated liposarcoma. Histopathological analyses, therefore, remain crucial in establishing a definitive diagnosis and guiding treatment decisions. The diverse MRI findings in this case, coupled with the observed histological variations within the same tumor entity, highlight the importance of cautious interpretation of imaging results and underscore the need for a comprehensive approach to well-differentiated orbital liposarcomas, integrating clinical presentation, imaging features, and detailed pathological evaluation.
